# Combined flap with Masquelet technique and 3D-printed titanium cage for reconstruction of traumatic composite heel defects: a case report and literature review

**DOI:** 10.3389/fsurg.2026.1798468

**Published:** 2026-05-07

**Authors:** Junhong Chen, Xiaojun Yu, Xulin Zhang, Qingshan Li, Zhiqiang Wang

**Affiliations:** 1 Department of Orthopaedics, Suining Central Hospital, Suining, China; 2 Department of Hand Microsurgery, Suining Central Hospital, Suining, China

**Keywords:** 3D-printed titanium cage, bone defect, flap reconstruction, Masquelet technique, open calcaneal fracture, soft tissue defect

## Abstract

**Background:**

Open calcaneal fractures accompanied by substantial segmental bone loss and severe soft-tissue damage represent a rare clinical presentation. The intricate anatomy of the heel region poses a significant challenge to limb-salvage efforts in such cases, for which a standardized treatment protocol remains to be established.

**Case summary:**

Here we report a case of extensive soft tissue defects around the ankle and heel, combined with a large segmental defect of the calcaneus, managed through a staged surgical approach. The first stage involved emergency debridement and coverage with antibiotic-loaded cement, while the residual heel skin was banked in the anterolateral thigh region. In the second stage, to address the composite soft tissue defects across multiple planes, a pedicled flap was combined with a free flap for reconstruction: a propeller flap based on a perforator of the peroneal artery was used to cover the posterior heel and lateral malleolus, followed by a free anterolateral thigh flap, which incorporated the banked skin, to resurface the heel and remaining critical areas. Split-thickness skin grafts were applied to non-critical zones. Antibiotic cement was implanted in the bone defect to prepare the site for later reconstruction. In the third stage, after confirming satisfactory soft tissue healing without signs of infection, a custom 3D-printed titanium cage prosthesis was implanted. To our knowledge, no similar case has been reported in the literature. This study represents the first application of a combined flap technique, the Masquelet induced membrane technique, and a 3D-printed patient-specific calcaneal prosthesis for this type of injury. One-year follow-up revealed well-positioned prosthesis on radiographic imaging, with acceptable foot contour and range of motion meeting the patient's basic functional needs. The American Orthopedic Foot & Ankle Society (AOFAS) score was 90 and the Maryland Foot Score was 89. The patient reported mild heel pain during weight-bearing, with minimal impact on daily activities.

**Conclusion:**

This case demonstrates that integrating the anti-infection benefits of the Masquelet technique, the precise structural support of 3D-printed titanium cages, and the revascularization advantages of microsurgery provides a promising combined strategy for achieving both aesthetic and functional reconstruction in complex composite heel defects.

## Introduction

The calcaneus is the most common site of foot fractures, accounting for approximately 75% of all foot fractures (1, 2). However, open calcaneal fractures are uncommon, representing about 10% of all calcaneal fractures (3). Among these, cases involving severe bone and soft tissue loss are particularly rare and present a significant clinical challenge, constituting a serious reconstructive problem. Gustilo type IIIB calcaneal fractures are high-energy injuries that frequently result in extensive bone loss, severe soft tissue damage, and a high risk of chronic osteomyelitis (4). When associated with degloving injuries, the blood supply to the subcutaneous tissue is further compromised, and concomitant tendon, nerve, and vascular injuries exacerbate soft tissue viability issues, substantially increasing the risk of infection and necrosis (5). The goals of treatment include achieving durable soft tissue coverage, controlling infection, restoring skeletal continuity and stability, and ultimately maximizing functional recovery of the foot.

Successful management of such complex injuries relies on a staged, multidisciplinary approach that integrates multiple techniques. In soft tissue reconstruction, when defects are extensive or involve multiple functional planes, a single flap often cannot achieve complete coverage. Combined flap techniques, particularly the integration of pedicled and free flaps, offer a viable solution for the precise repair of such composite defects. The peroneal artery perforator propeller flap is a reliable option for reconstructing the perimalleolar region, while the free anterolateral thigh flap—with its abundant blood supply, large surface area, and long vascular pedicle—is commonly used to cover extensive heel defects. Current strategies for bone defect reconstruction include vascularized fibular grafts, Ilizarov bone transport, the Masquelet induced membrane technique, and the implantation of 3D-printed prostheses (6). While the Ilizarov technique is widely applicable, it is associated with a prolonged treatment duration, high care burden, and risks such as pin-tract infection, joint stiffness, and nerve injury (7). The Masquelet technique alone is relatively straightforward and time-controlled but does not permit early weight-bearing in cases of large segmental defects. Moreover, difficulties in cement shaping and removal may damage the induced membrane, potentially compromising graft integration and increasing infection risk (6). Conventional custom prostheses are often limited by suboptimal host-bone matching and high postoperative infection rates. The simultaneous optimization of structural and functional reconstruction in such composite bone and soft tissue defects remains a major challenge in orthopaedic and microsurgical reconstruction. In recent years, the introduction of 3D printing technology combined with the Masquelet technique has brought new breakthroughs to this field (8). Through preoperative precise design and fabrication of porous titanium alloy prostheses that closely match the bone defect morphology, anatomical reconstruction of the calcaneus can be achieved while providing good initial mechanical stability, offering a novel approach for individualized functional restoration.

This study reports a case of traumatic composite heel defect successfully treated with a combined strategy of “integrated flaps, the Masquelet technique, and a 3D-printed patient-specific titanium cage prosthesis.” We detail the surgical planning, technical considerations, and postoperative outcomes of this integrated approach. By reviewing relevant literature, we further discuss the advantages, potential challenges, and clinical prospects of this comprehensive treatment strategy for such severe injuries, aiming to provide clinicians with new insights and a reference framework for managing similarly complex cases.

## Case report

A 59-year-old male patient presented with left foot pain and limited mobility after being struck by a heavy object, accompanied by foot valgus deformity, paresthesia on the dorsal aspect of the toes, and extensive soft-tissue defects in the heel and ankle region (approximately 25 cm × 15 cm). CT scan of the left foot revealed a comminuted fracture of the left calcaneus with substantial bone loss, multiple fractures of the medial and lateral malleoli, talus, navicular bone, cuboid bone, and base of the fifth metatarsal, as well as suspected linear fractures at the distal ends of the middle phalanges of the third and fourth toes. The findings were consistent with a Sanders type IV calcaneal fracture accompanied by extensive bone loss.

**Figure 1:**
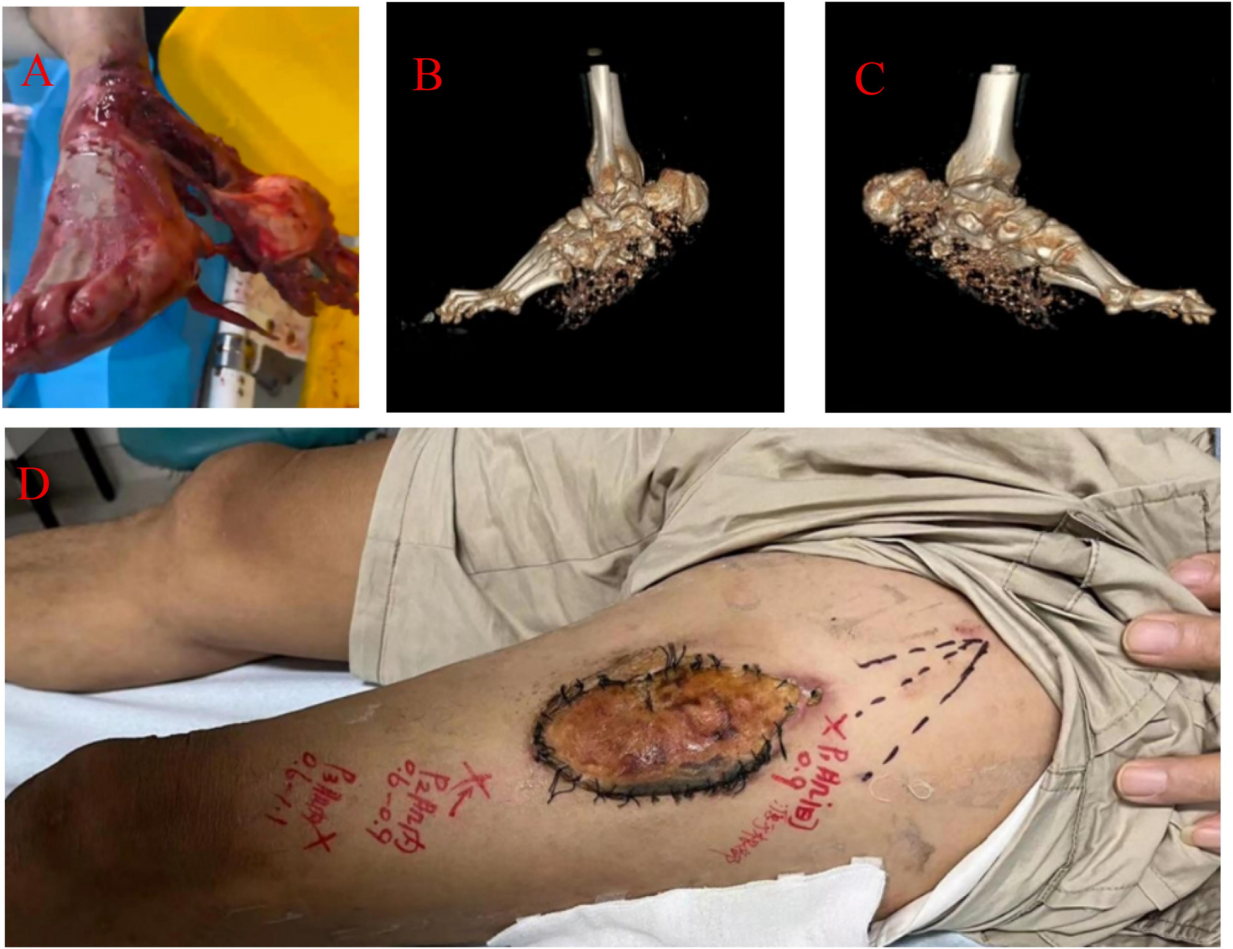
**(A–C)** preoperative presentation of the left foot and three-dimensional computed tomography imaging demonstrate a comminuted fracture of the calcaneus with extensive bone loss, alongside substantial soft-tissue defects involving the medial and lateral malleoli and the plantar region, with only a remnant of heel skin preserved. **(D)** Intraoperative view showing the temporary transfer and fixation of the preserved heel skin as a “parasitic” flap to the thigh.

A first-generation cephalosporin was administered intravenously 30 min preoperatively for infection prophylaxis. Intraoperative exploration showed large-area degloving injuries involving the medial, lateral, posterior ankle, and mid-posterior plantar regions; complete rupture of the posterior tibial artery; approximately 50% transection of the tibial nerve; tear and loss of the medial ankle ligament complex; severe contusion of the flexor hallucis longus, flexor digitorum longus, and tibialis posterior tendons; and extensive damage to the plantar muscle group. Approximately 50% of the calcaneal body was lost, with about 60% of its lateral articular surface being free and fragmented. Additionally, comminuted fractures with free bone fragments were observed in the medial and lateral malleoli, talus, navicular, cuboid, and fifth metatarsal bones. The subtalar joint was completely dislocated. All non-viable and heavily contaminated tissues along with free bone fragments were debrided. Limited internal fixation using K-wires was applied to the remaining calcaneal fragments to maintain the general contour, and antibiotic-loaded bone cement was implanted into the bone defect area as a spacer. A vacuum-sealing drainage (VSD) device was placed over the wound. The degloved skin from the heel region, which had questionable vascularity, was harvested as a full-thickness skin graft, thinned, and temporarily sutured to the lateral aspect of the ipsilateral mid-thigh for potential use in a subsequent procedure (Figure 1).

### Second surgery (day 6 post-emergency surgery)

**Figure 2:**
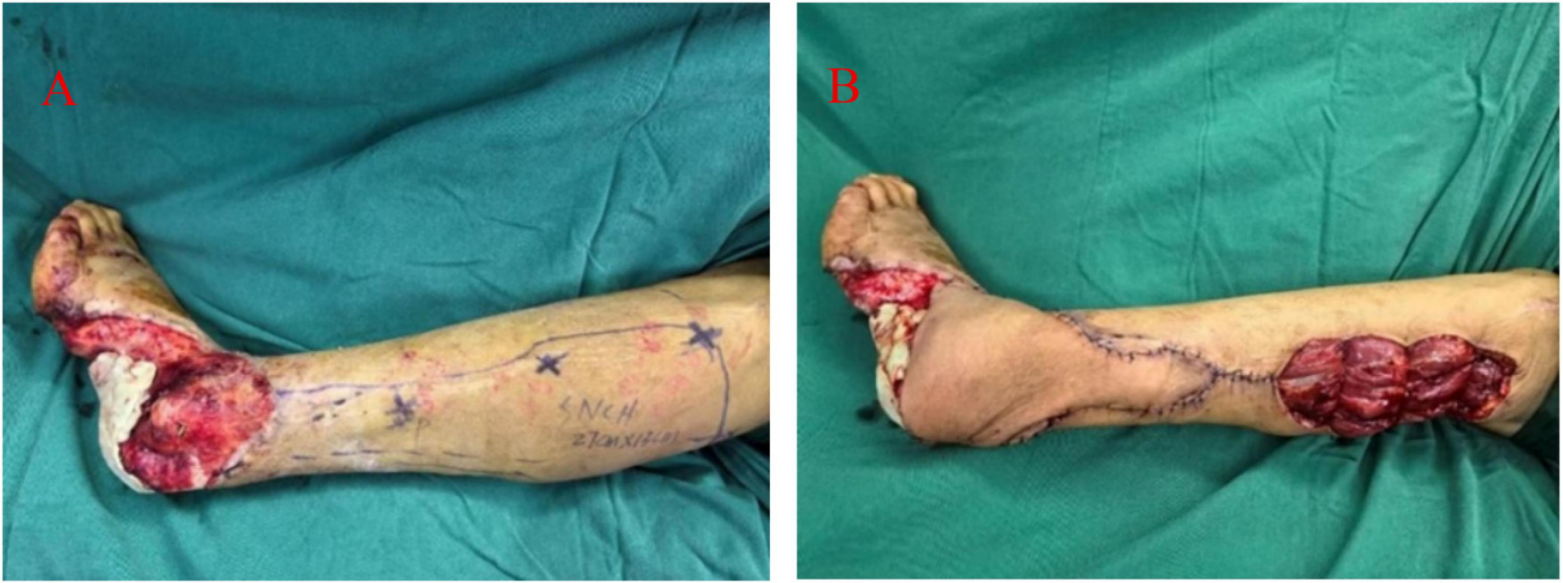
The medial ankle and heel defect was reconstructed using a peroneal artery perforator-based propeller flap during the second-stage procedure. **(A)** Preoperative view. **(B)** Postoperative view.

The patient exhibited no signs of systemic or local infection in the left foot. Laboratory tests showed a white blood cell (WBC) count of 7.24 × 10^9^/L and a C-reactive protein (CRP) level of <1.2 mg/L, meeting the criteria for surgery. The primary goal of this stage was to reconstruct the composite soft tissue defect involving the heel and lateral malleolus. A posterolateral sural fasciocutaneous flap, based on perforators from the peroneal artery, was designed over the ipsilateral calf according to the size and contour of the left posterolateral foot defect. Intraoperatively, the dominant perforator was meticulously identified and isolated. The vascular pedicle was sufficiently skeletonized to ensure an adequate rotation arc for tension-free coverage of the recipient site. The flap was transferred to the recipient area without tension. After confirming robust perfusion, as evidenced by active bleeding from the edges and a pink color, the flap was inset using absorbable sutures in a layered fashion. The donor site was partially closed primarily following subcutaneous undermining. The remaining wound that could not be approximated was covered with a vacuum-sealing drainage (VSD) system. The affected limb was elevated using an external fixation frame. This setup effectively prevented pressure on the flap while maintaining the ankle joint in a functional position. The bone defect in the heel region remained temporarily filled with antibiotic-loaded bone cement to preserve space for subsequent bone reconstruction (Figure 2).

### The third surgery (7 days after the second procedure)

**Figure 3:**
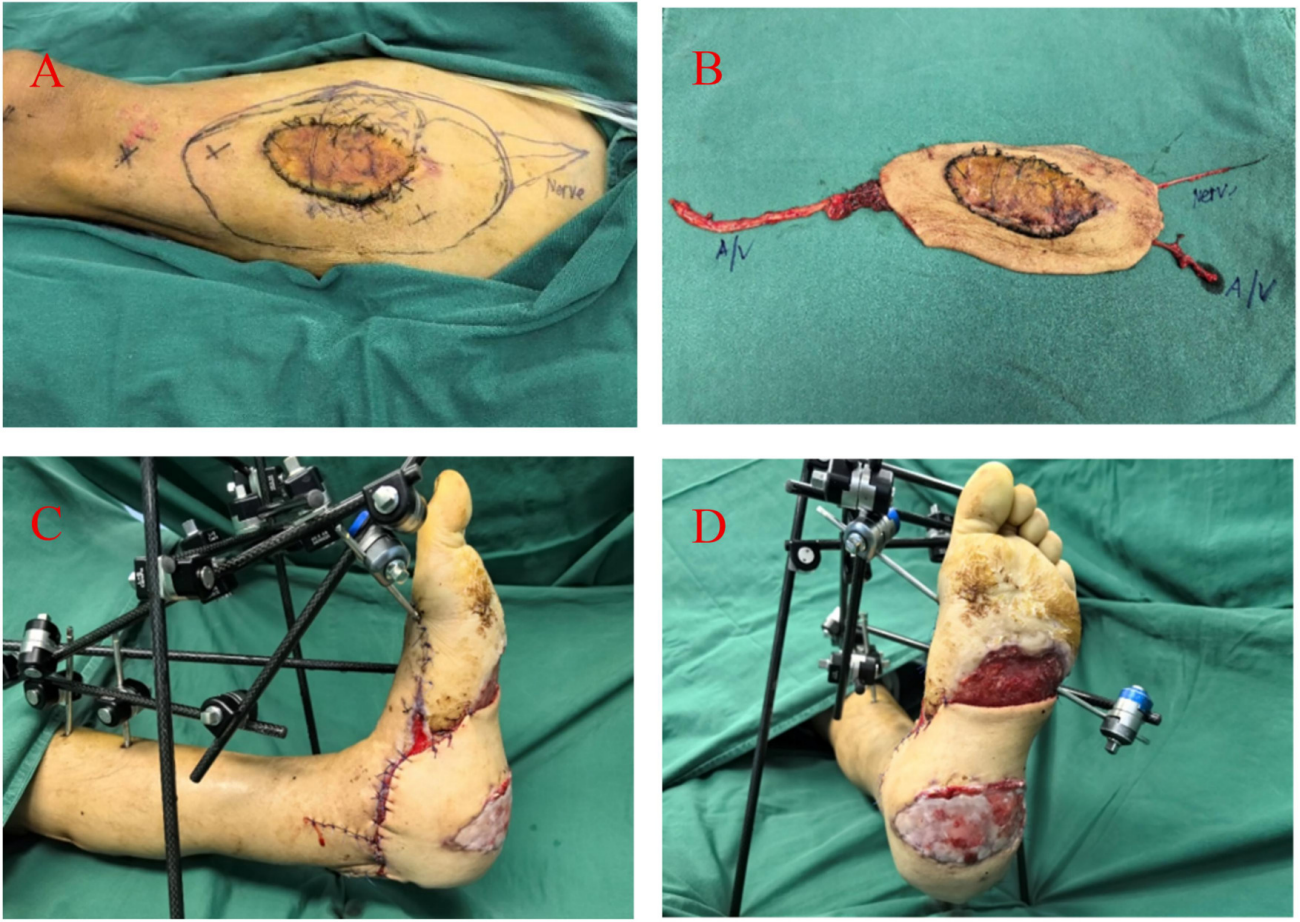
Reconstruction of the heel, plantar, and medial ankle regions using a pedicled anterolateral thigh free flap with preserved heel skin. **(A)** Preoperative flap design and the well-preserved status of the heel skin flap. **(B)** Harvesting of the anterolateral thigh free flap along with the transferred heel skin. **(C,D)** Post-transplantation fixation of the flap using an external fixator.

It involved removal of the left foot external fixator and VSD dressing. The wound showed satisfactory granulation tissue growth and well-controlled inflammation, after which the antibiotic-impregnated bone cement was removed. The anterolateral thigh flap was designed and harvested from the ipsilateral thigh according to the size and shape of the foot defect. Intraoperative exploration confirmed preservation of at least two perforators originating from the descending branch of the lateral circumflex femoral artery. The vascular pedicle and the lateral femoral cutaneous nerve were carefully dissected and prepared. The flap was thinned meticulously to optimize contour and reduce bulk at the recipient site. At the recipient site on the left ankle or dorsum of the foot, the anterior tibial artery (one), two accompanying veins, and one available sensory nerve were dissected and exposed. Under microscopic guidance, end-to-end anastomoses were performed between the flap arteries, veins, and nerve and their corresponding recipient vessels and nerve. After release of the vascular clamps, the flap appeared well-perfused with rosy color, appropriate tension, and favorable capillary refill. Once stable blood supply to the flap was confirmed, it was sutured in place to cover key weight-bearing and critical regions including the heel and posterior ankle. A split-thickness skin graft was harvested from the ipsilateral thigh, trimmed, and transplanted to cover the remaining wounds on the plantar surface, lateral foot, and posterior calf. According to the anatomical requirements, the plantar and lateral foot areas were dressed with a tie-over bolster, while the posterior calf was covered and secured with a VSD. Postoperatively, the affected limb was immobilized with an external fixator and elevated to facilitate venous return of the flap and survival of the skin graft (Figure 3).

### The fourth surgery (60 days after the third procedure)

**Figure 4:**
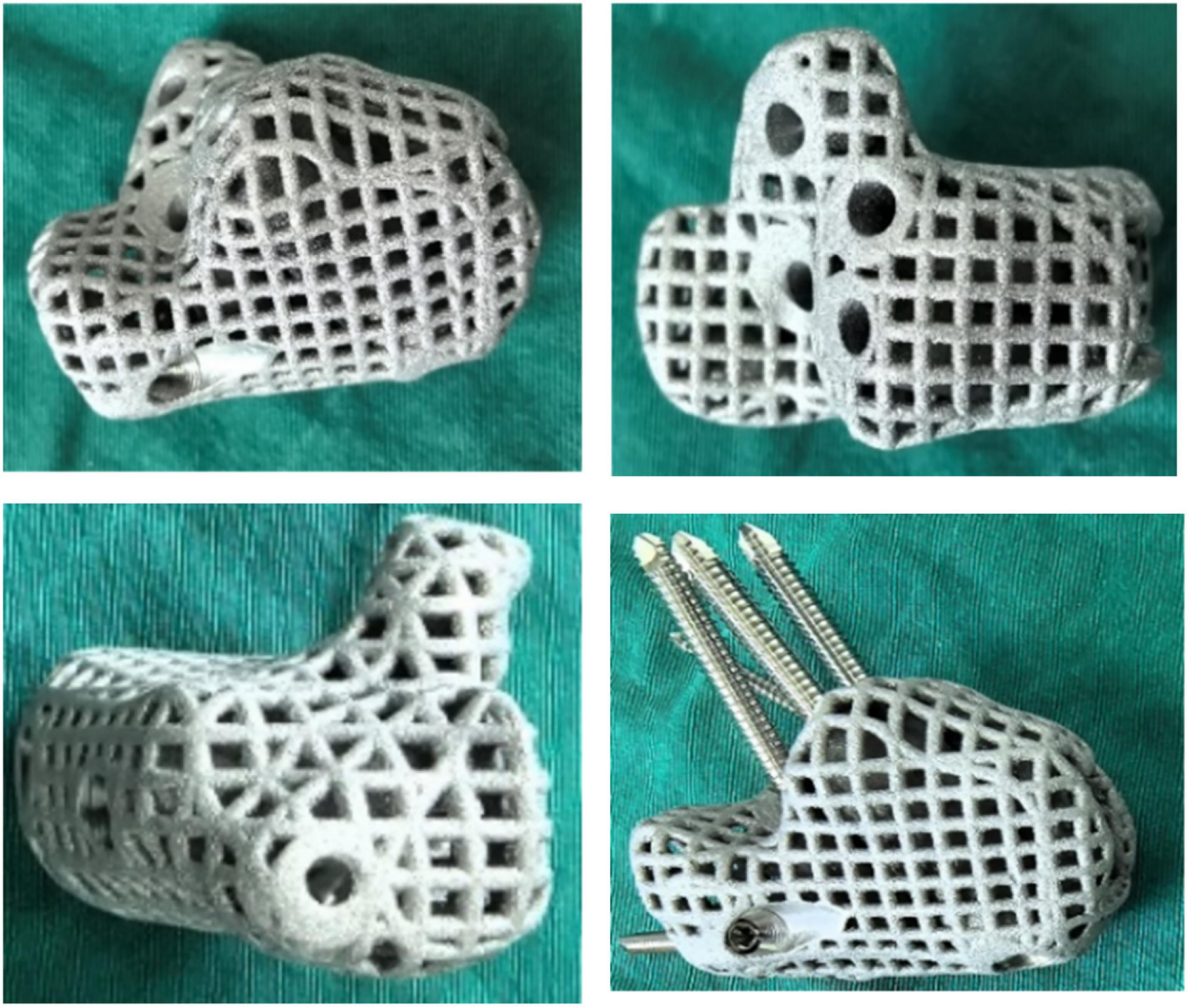
Titanium cage design and fixation technique. The cage, filled with both autogenous and allogeneic bone graft material, is augmented with supplementary suture fixation to enhance stability and potentially improve its functional longevity.

**Figure 5:**
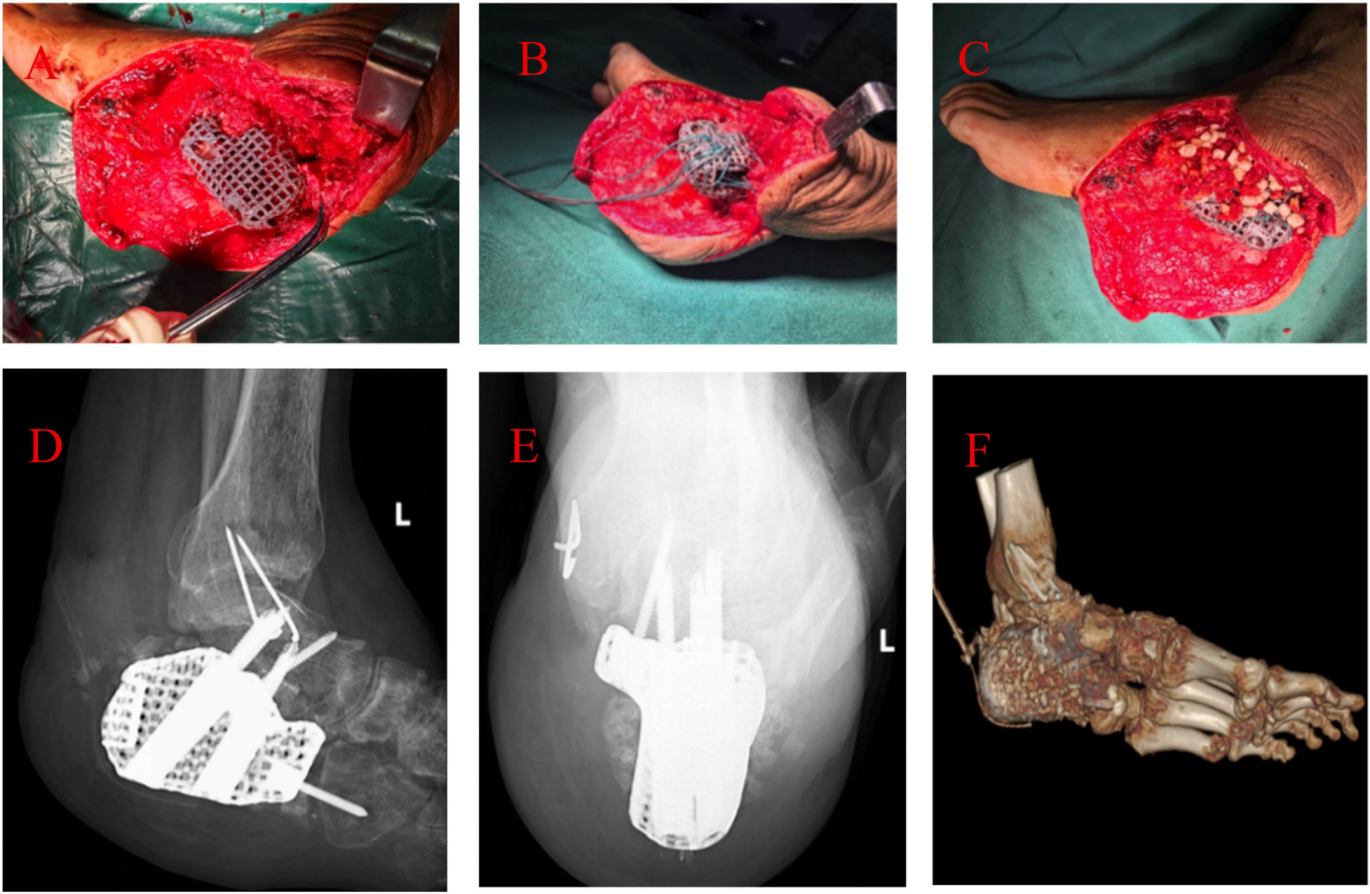
Surgical reconstruction of the calcaneal defect. **(A)** Implantation of the titanium cage. **(B)** Intraoperative fixation of the titanium cage. **(C)** Placement of bone graft material within and around the titanium cage. **(D–F)** Postoperative radiographic and CT images demonstrating the reconstruction outcome.

It was performed following preoperative assessment showing no signs of local infection, with a white blood cell (WBC) count of 7.24 × 10^9^/L and C-reactive protein (CRP) level <1.2 mg/L. Intraoperative frozen section analysis of periprosthetic tissue revealed fewer than 5 neutrophils per high-power field (HPF), confirming no evidence of active infection, and thus bone reconstruction was proceeded. The antibiotic-loaded bone cement was carefully removed using osteotomes and curettes to fully expose the calcaneal defect, and the temporary K-wires were extracted. The wound was thoroughly debrided with pulsed lavage using 3% hydrogen peroxide, normal saline, and diluted povidone-iodine solution. A tricortical iliac bone graft (approximately 4 × 5 × 2 cm) was harvested from the ipsilateral anterior superior iliac spine, morcellized into granules, and mixed with an osteoinductive bone substitute material. A preoperatively designed 3D-printed custom titanium prosthesis (titanium cage), mirroring the contralateral calcaneus, was accurately implanted into the defect. After provisional fixation with K-wires, intraoperative multi-plane fluoroscopy with a C-arm confirmed satisfactory prosthesis positioning, alignment, and subtalar joint congruency. The Achilles tendon insertion was released and dissected, then securely re-attached to the pre-drilled holes on the posterosuperior aspect of the prosthesis using suture anchors, thereby reconstructing the Achilles tendon-prosthesis continuity. The soft tissues were closed in layers over the prosthesis. A short-leg plaster splint was applied to immobilize the ankle in the functional position (Figures 4, 5).

**Figure 6:**
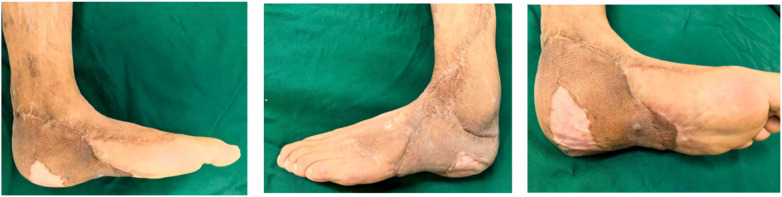
Postoperative clinical appearance of the foot at 6 months.

During the 18-month postoperative follow-up, the reconstructed heel showed no evidence of flap necrosis or ulceration. The patient achieved partial weight-bearing with crutch assistance at 3 months and progressed to full weight-bearing ambulation by 6 months postoperatively (Figure 6).

## Discussion

Open calcaneal fractures complicated by segmental bone defects and severe soft tissue injury resulting from high-energy trauma represent one of the most challenging dilemmas in orthopedic trauma surgery. The unique structural and biomechanical functions of the calcaneus underscore the complexity of its reconstruction: the heel soft tissue is thin and poorly vascularized, making coverage difficult in the event of a defect; as a key component of the foot arch, the calcaneus's length, height, and articular angles are critical to foot function (9). Moreover, severe contamination and the high-energy nature of the injury predispose to deep infection, further increasing the risk of limb failure (10). The success in this case lies in the integration of three core techniques into a staged, synergistic comprehensive reconstruction strategy.

For segmental bone defects, traditional treatment strategies primarily include vascularized fibular grafting, open bone grafting (the Papineau technique), and bone transport. However, given the irregular morphology of the calcaneus, these techniques often fall short in achieving precise anatomical reconstruction, and long-term functional recovery of the affected limb tends to be suboptimal (11). In the present case, although amputation would offer a relatively straightforward procedure and a shorter rehabilitation period, the patient—a middle-aged male with high demands for both ambulatory function and aesthetic appearance, and with preserved soft tissue and function in the forefoot—made limb salvage a preferable option in pursuit of better functional outcomes. Autogenous iliac crest or fibular grafting has traditionally been regarded as the gold standard for reconstructing bone defects. However, for the large calcaneal defect in this case, which accounted for 50% of the bone volume, isolated cancellous bone grafting lacked adequate structural support. It would be unable to withstand the tensile forces exerted by the Achilles tendon in the early postoperative period, nor tolerate weight-bearing loads in the later phase, predisposing to bone resorption, collapse, and even malunion. Vascularized fibular flap grafting has been established as an effective method for treating large segmental defects in the extremities (12). Nevertheless, its application in this case presented significant limitations. First, this case involved extensive soft tissue defects around the ankle and heel. Harvesting a chimeric fibular flap for combined bone and soft tissue reconstruction would entail substantial donor-site morbidity, while the recipient site would require simultaneous management of a composite bone and soft tissue defect—rendering the procedure technically demanding and making it difficult to achieve both adequate bone reconstruction and soft tissue coverage. Furthermore, the fibular flap is less precise in terms of mechanical matching compared with customized prostheses. For severe Gustilo type III open fractures, primary internal fixation or prosthetic implantation carries a prohibitively high risk of infection (13). In this case, the soft tissue condition was extremely poor at the time of the initial debridement, with severe contamination; prosthetic implantation at that stage would risk devastating consequences, including implant removal, reconstruction failure, or even amputation in the event of infection.

Based on the above analysis, no single technique could address all the challenges in this case. The combination of a perforator flap technique enabled complex soft tissue coverage in multiple planes and addressed weight-bearing requirements of the heel; the Masquelet technique provided infection control and facilitated osseointegration; and the 3D-printed titanium cage allowed precise anatomical reconstruction of the calcaneus with early mechanical stability. The sequential integration of these three modalities reflects a rigorous logic—from biological preparation to mechanical reconstruction—in managing such extreme limb salvage cases.

The management of combined calcaneal and perimalleolar soft tissue defects demands not only simultaneous wound coverage and skeletal reconstruction but also places high demands on postoperative functional recovery. In this case, a satisfactory outcome was achieved through a staged surgical strategy. During the second-stage procedure, a peroneal artery perforator-based propeller flap and an anterolateral thigh (ALT) free flap were employed to reconstruct the heel and perimalleolar defects, respectively. This approach established a durable, weight-bearing soft tissue envelope, partially restored foot contour and protective sensation, and created favorable conditions for subsequent bone reconstruction. Building on this foundation, the third-stage surgery successfully reconstructed the calcaneal defect using the Masquelet technique combined with a 3D-printed, patient-specific titanium cage. Postoperative follow-up demonstrated excellent restoration of both foot appearance and function, confirming the therapeutic potential of this combined strategy for such complex injuries.

With advances in microsurgery, options for repairing perimalleolar soft tissue defects have diversified. Pedicled flaps—such as the sural neurocutaneous flap, posterior tibial artery perforator flap, lateral supramalleolar flap, and medial plantar flap—as well as free flaps, including the ALT flap, superficial circumflex iliac artery perforator (SCIP) flap, and deep inferior epigastric artery perforator (DIEP) flap, have all found clinical application (14–18). While pedicled or local flaps from the lower leg and foot offer simplicity, obviate the need for vascular anastomosis, and yield reliable outcomes, they are associated with significant donor-site morbidity, limited coverage area, and risks such as partial flap necrosis due to vascular torsion or insufficient perfusion—particularly venous congestion in retrograde flaps, which can severely compromise lower leg aesthetics (19). In contrast, free flaps like the ALT, DIEP, and SCIP flaps offer greater flexibility and consistent perforators but entail longer operative times, requiring two surgical teams, and demand precise microsurgical technique for vascular anastomosis. Furthermore, factors such as the relative bulkiness of DIEP and ALT flaps due to subcutaneous fat, the small caliber of the SCIP flap pedicle, and potential donor-site pigmentation often influence flap selection (20).

In this case, employing a single flap to cover such an extensive defect would have carried high risks of infection, excessive bulk, and necrosis. Therefore, we innovatively utilized the patient's preserved heel skin as a “parasitic” flap, pre-positioned in the anterolateral thigh region, and employed a staged combination of a peroneal artery perforator propeller flap and an ALT flap for soft tissue reconstruction. Zhang (21) described prefabricating a heel flap by deepithelializing and thinning avulsed heel skin, grafting it onto the fascia lata of the anterolateral thigh, and performing free vascularized transplantation back to the original site two weeks later, achieving favorable clinical results. The strategy adopted here offers several advantages: First, it decomposes a complex multiplanar defect into several uniplannar defects, allowing sequential repair. This avoids the excessive bulk associated with using a single large flap and reduces the technical difficulty and complication risks inherent in complex chimeric or combined flaps. Second, the flaps cover critical areas, while the remaining regions can be managed with skin grafts—a zonal repair approach difficult to achieve with a single combined flap, thereby facilitating postoperative shoe wear and functional recovery. Third, it establishes a dual blood supply system, significantly enhancing flap survival in the notoriously challenging vascular environment of the heel. Additionally, by coapting the flap's nerve to the sural nerve, protective sensation was partially restored to the heel. Compared to traditional free flaps or extensive skin grafting, this combined flap technique minimizes donor-site morbidity by utilizing local or adjacent tissues, avoiding complications associated with distant donor sites. Moreover, successful soft tissue coverage laid the essential foundation for the subsequent application of the Masquelet technique, underscoring its critical role as emphasized by Morelli et al. (22).

The peroneal artery, a major blood supply to the lateral calf skin, originates from the proximal posterior tibial artery, descends along the interosseous membrane, and forms extensive anastomoses with the posterior tibial artery proximal to the ankle; its perforators also contribute to the anterior ankle vascular network (23, 24). In this case, the peroneal artery perforator propeller flap was chosen to cover the lateral malleolus, Achilles tendon, and retrocalcaneal regions. This flap offers reliable blood supply and can incorporate the lateral sural cutaneous nerve branch to restore sensation, helping prevent ulcers and chronic infection. Its texture and thickness are well-matched to the recipient site, facilitating shoe wear, and donor-site morbidity is minimal (25). The ALT free flap, based on perforators from the descending branch of the lateral circumflex femoral artery, is highly versatile. It can be tailored as a flow-through, bilobed, chimeric, thinned, or conjoined flap according to defect requirements (26). It can include vastus lateralis muscle to fill dead space, promote bone healing, and enhance resistance to infection (27). Its fascia lata component can be used for ligament reconstruction, aiding early functional recovery. The flap provides ample tissue to cover large defects, potentially obviating the need for multiple vascular anastomoses and thus reducing surgical complexity and risk (28). Its vascular caliber matches well with recipient vessels in the foot and ankle, resulting in a low incidence of vascular complications and high survival rates (29). It also allows for sensory reinnervation via the lateral femoral cutaneous nerve and has minimal impact on donor-site function. Limitations include potential impairment of hip flexion if a large muscle component is harvested, poor donor-site aesthetics often requiring skin grafting for large defects, and excessive bulk in the recipient site if significant muscle is included, frequently necessitating secondary debulking.

With ongoing advances in microsurgery, bone and soft tissue engineering, and biomaterials, the Masquelet technique has seen increasingly widespread clinical application. Through refinements of the technique itself and its combination with other methods, significant progress has been made in repairing large segmental bone defects (30). The core of this technique involves implanting a PMMA bone cement spacer into the bone and soft tissue defect to induce the formation of a bioactive membrane. This membrane not only occupies dead space, covers the wound, and provides temporary mechanical support but also effectively prevents fibrous tissue ingrowth, creating a favorable biological microenvironment for subsequent bone grafting (31). However, the Masquelet technique combined with particulate bone grafting alone is insufficient for precisely reconstructing the complex three-dimensional anatomy of the calcaneus or providing a robust mechanical insertion point for the Achilles tendon (32). Similarly, traditional pedicled bone grafting or the Ilizarov bone transport technique often fail to achieve accurate anatomical restoration of the calcaneus, frequently resulting in suboptimal therapeutic outcomes (33).

Therefore, this study combined a 3D-printed patient-specific titanium cage with the Masquelet technique to reconstruct the calcaneal defect. This combined strategy enables precise anatomical restoration of the calcaneus while providing a stable foundation for overlying soft tissues. The custom-designed titanium cage, based on imaging data from the contralateral healthy calcaneus, features a mesh structure with a smooth surface to minimize wear. Its internal design, devoid of crossbeams, effectively maintains calcaneal shape, analogous to the use of spinal titanium cages for reconstructing segmental vertebral defects (34, 35). The implant includes an anatomical groove corresponding to the calcaneal tuberosity, which not only facilitates bone ingrowth and reduces long-term failure risk but also allows for early stable fixation via sutures and screws, thereby mitigating risks of infection, flap breakdown, and systemic complications. Preoperatively precise planning ensures the cage perfectly matches the bone defect, providing inherent mechanical stability and aiding in deformity correction. Its porous structure facilitates ligament attachment, supporting postoperative functional recovery. Combining 3D-printed titanium cages with the Masquelet-induced membrane technique for calcaneal reconstruction ensures the quality and dimensions of the induced membrane, offering both technical simplicity and biomechanical advantages (6). Chou (36) reported a case of total calcanectomy for osteosarcoma managed with a custom calcaneal prosthesis, where the patient experienced only mild plantar and heel pain, returned to work within six months, and showed no signs of foot instability over a 36-month follow-up. Park (37) implanted a 3D-printed titanium calcaneal prosthesis in a 23-year-old patient with an aggressive fibromatosis; at 16 months postoperatively, the patient achieved gait without a limp, with only minor early discomfort. For extensive calcaneal defects, traditional bone cement spacers often require staged surgery for removal and bone grafting, significantly prolonging rehabilitation. In contrast, Jungo (38) used a 3D-printed titanium alloy prosthesis to reconstruct a defect following total calcanectomy, demonstrating good weight-bearing capacity and biocompatibility of the material, though its long-term efficacy requires further validation.

This study has limitations, being a single case report with a short follow-up duration. The long-term outcomes—such as prosthetic loosening, wear, and the durability of osseointegration—warrant further validation through extended follow-up and accumulation of additional cases.

## Conclusion

The successful management of extensive calcaneal defects with concomitant severe soft-tissue injury hinges on the simultaneous restoration of both wound coverage and skeletal integrity to re-establish foot structure and function. In this study, a staged surgical approach was adopted. Initial soft-tissue reconstruction was achieved using a peroneal artery perforator propeller flap and a subsequent free anterolateral thigh flap. This strategy provided a durable, weight-bearing soft-tissue envelope for the heel while restoring protective sensation, thereby establishing a favorable biological milieu for definitive bone reconstruction. Subsequently, the osseous defect was successfully addressed by integrating the Masquelet induced-membrane technique with the implantation of a patient-specific, 3D-printed titanium cage. Postoperatively, the patient demonstrated satisfactory restoration of foot contour and functional outcomes.

## Data Availability

The original contributions presented in the study are included in the article/Supplementary Material, further inquiries can be directed to the corresponding author.

## References

[1] Spierings KE Min M Nooijen LE Swords MP Schepers T. Managing the open calcaneal fracture: a systematic review. Foot Ankle Surg. (2018) 25(6):707–13. 10.1016/j.fas.2018.10.005 30467055

[2] Firoozabadi R Kramer PA Benirschke SK. Plantar medial wounds associated with calcaneal fractures. Foot Ankle Int. (2013) 34(7):941–8. 10.1177/1071100713481460 23478886

[3] Zhao WG Zhang YZ. Comparison and predictive factors analysis for efficacy and safety of Kirschner wire, anatomical plate fixation and cannulated screw in treating patients with open calcaneal fractures. Medicine (Baltimore). (2019) 98(43):e17498. 10.1097/MD.0000000000017498 31651853 PMC6824657

[4] Torre A Monti MD. Osteomyelitis of the calcaneus with pathologic fracture. J Foot Ankle Surg. (2020) 59(3):641. 10.1053/j.jfas.2019.09.022 32354522

[5] Chen ZB Cong XB Zhou P. Management of foot degloving injury with bilateral anterolateral thigh flaps. Trauma Case Rep. (2020) 29:100337. 10.1016/j.tcr.2020.100337 32875047 PMC7451611

[6] Zhang LF Lu CY Wang XH Guo SY Zhang HL Lv YQ. 3D printing assisted Masquelet technique in the treatment of calcaneal defects. J Trauma Acute Care Surg. (2021) 23(7):507–11. 10.1111/os.12873 PMC8126905 33768676

[7] Ma LJ Sun GW. Application of Ilizarov external fixator in orthopedics. Med Recapit. (2020) 26(2):5. 10.5035/nishiseisai.50.969

[8] Canavese F Dimeglio A Bonnel F. Postoperative CT-scan 3D reconstruction of the calcaneus following lateral calcaneal lengthening osteotomy for flatfoot deformity in children. Is the surgical procedure potentially associated with subtalar joint damage? Foot Ankle Surg. (2018) 24(5):453–9. 10.1016/j.fas.2017.05.005 29409196

[9] Brinker MR Loncarich DP Melissinos EG O'Connor DP. Calcaneogenesis. J Bone Joint Surg Br. (2009) 91(5):662–5. 10.1302/0301-620X.91B5.21938 19407304

[10] Mehta S Mirza AJ Dunbar RP Barei DP Benirschke SK. A staged treatment plan for the management of type II and type IIIA open calcaneus fractures. J Orthop Trauma. (2010) 24(3):142–7. 10.1097/BOT.0b013e3181b5c0a4 20182249

[11] Hamiti Y Yushan M Lu C Yusufu A. Reconstruction of massive tibial defect caused by osteomyelitis using induced membrane followed by trifocal bone transport technique: a retrospective study and our experience. BMC Surg. (2021) 21(1):419. 10.1186/s12893-021-01421-x 34911504 PMC8672610

[12] Liu R Li Z Jin A. Heel reconstruction with parallel fibular osteoseptocutaneous flap. Acta Orthop Belg. (2016) 82(2):275–9. 27682289

[13] Williams CG Coffey MJ Shorten P Lyions JD Laughlin RT. Staged subtalar fusion for severe calcaneus fractures with bone loss. Open Orthop J. (2013) 7:614–8. 10.2174/1874325001307010614 24339843 PMC3849750

[14] Yu CQ Liu Y Wang JL Zheng H Guo J Sui ZQ. Anatomical study and clinical application of cross-donor flaps pedicled with peroneal vessels. Chin J Microsurg. (2022) 45(1):6. 10.3760/cma.j.cn441206-20211013-00240

[15] Wang J Hu Y Zhang XJ Xu XQ Liu HC Li ZD. Analysis of the efficacy of posterior tibial artery musculocutaneous perforator flap in repairing skin and soft tissue defects in the ankle and foot. Chin J Bone Joint Inj. (2022) 37(4):432–4. (in Chinese)

[16] Sun Y Yang LX Qiu YF Huang XX Wang GL Zhu W Exploring the use of perforating propeller flaps in the repair of soft tissue defects around the ankle based on the free-style concept. J Trauma Surg. (2022) 24(1):6. (in Chinese)

[17] Dong L Liang OC Xin Z Gen ZY Lin L Hui WG. Comparative study of anterolateral thigh perforator flap and deep inferior epigastric perforator flap in repairing large area soft tissue defects of lower limbs. J Trauma Surg. (2021) 23(6):412–6. 10.3969/j.issn.1009-4237.2021.06.003

[18] Yu T Lan RY Jiang JY Pi JB Guan RZ Yu YZ. Microscope-assisted thinning of inferior epigastric artery perforator flap in repairing large skin and soft tissue defects of limbs. J Trauma Surg. (2021) 23(6):4. 10.3969/j.issn.1009-4237.2021.06.005

[19] Fan AM Chen Y Li Z Xie JF He Z Wang SF. Free anterolateral thigh perforator flap for repairing soft tissue defect on the foot and ankle. Orthop J China. (2022) 30(10):3. (in Chinese)

[20] Kitaoka HB Alexander IJ Adelaar RS Nunley JA Myerson MS Sanders M. Clinical rating systems for the ankle-hindfoot, midfoot, hallux, and lesser toes. Foot Ankle Int. (1994) 15(7):349–53. 10.1177/107110079701800315 7951968

[21] Zhang M Guo A Zhang GL. Repair of heel soft tissue avulsion injury with pre fabricated skin flap for heel skin care. Chin J Microsurg. (2010) 33(5):404–5. 10.3760/cma.j.issn.1001-2036.2010.05.018

[22] Morelli I Drago L George DA Gallazzi E Scarponi S Romanò CL. Masquelet technique: myth or reality? A systematic review and meta-analysis. Injury. (2016) 47:S68–76. 10.1016/S0020-1383(16)30842-7 28040090

[23] Geddes CR Morris SF Neligan PC. Perforator flaps: evolution, classification, and applications. Ann Plast Surg. (2003) 50(1):90–9. 10.1097/01.SAP.0000032309.30122.55 12545116

[24] Jakubietz RG Jakubietz MG Gruenert JG Kloss DF. The 180-degree perforator-based propeller flap for soft tissue coverage of the distal, lower extremity: a new method to achieve reliable coverage of the distal lower extremity with a local, fasciocutaneous perforator flap. Ann Plast Surg. (2007) 59(6):667–71. 10.1097/SAP.0b013e31803c9b66 18046150

[25] Zhang XC Lu ZF Zhao HS Ou CL. Peroneal artery perforator propeller flap in repairing foot and ankle wounds. J Trauma Surg. (2025):27(3):221–4. 10.3969/j.issn.1009-4237.2025.03.011

[26] Qing XY Yu TJ Bo LY Bing ZZ Hua JC Xing Z Expert consensus on the anatomical characteristics and localization methods of perforators of anterolateral thigh flap (2024 edition). Chin J Clin Anat. (2024) 42(5):489–99. 10.13418/j.issn.1001-165x.2024.5.01

[27] Zhang YM Liu HR Zhang RH Chen YG Ma TP Yu ZY. Combined free anterolateral thigh flap and tibial bone transport for resurfacing large segmental bone and soft tissue defect of lower extremities. Chin J Traumatol. (2017) 33(2):129–33. 10.3760/cma.j.issn.1001-8050.2017.02.008

[28] Song DJ Li Z Zhang YX Zhou B Lv CL Tang YY. Application of expanded anterolateral thigh myocutaneous flap in the repair of huge chest wall defect. Chin J Reparative Reconstr Surg. (2022) 36(7):36. 10.7507/1002-1892.202202001 PMC9288917 35848179

[29] Fan AM Chen Y Li Z Xie JF He Z Wang SF. Free anterolateral thigh perforator flap for repairing soft tissue defect on the foot and ankle. Orthop J China. (2022) 30(10):3. (in Chinese)

[30] Abdeen A Hoang BH Athanasian EA Morris CD Boland PJ Healey JH. Allograft-prosthesis composite reconstruction of the proximal part of the humerus: functional outcome and survivorship. J Bone Joint Surg Am. (2010) 92:188–96. 10.2106/JBJS.H.00815 19797576

[31] Wang SQ Wang H Jia QY. Clinical efficacy analysis of membrane induction technology combined with free ultra-thin anterior lateral thigh perforator flap for repairing ankle wounds. Med J Chin PLA. (2025) 1(1):1–10. (in Chinese)

[32] Fan HB Fu J Li XD Pei YJ Li XK Pei GX Implantation of customized 3-D printed titanium prosthesis in limb salvage surgery: a case series and review of the literature. World J Surg Oncol. (2015) 13:308. 10.1186/s12957-015-0723-2 26537339 PMC4632365

[33] Zhao J Wang Z Long C He H Zhao W Zhang J. Using 3D printing-assisted shaping titanium cages and Masquelet techniques to reconstruct calcaneal osteomyelitis complicated by extensive soft tissue and uncontrolled defects. Injury. (2023) 54(10):110977. 10.1016/j.injury.2023.110977 37684116

[34] Fang T Zhang M Yan J Zhao JL Zhou Q. Comparative analysis of 3D-printed artificial vertebral body versus titanium mesh cage in repairing bone defects following single-level anterior cervical corpectomy and fusion. Med Sci Monit. (2021) 27:e928022. 10.12659/MSM.928022 33550326 PMC7876950

[35] Yu FB Miao JH Liao XY Wang XW Chen Y Chen DY. Evaluation of a new type of titanium mesh cage versus the traditional titanium mesh cage for single-level, anterior cervical corpectomy and fusion. Eur Spine J. (2013) 22(12):2891–6. 10.1007/s00586-013-2976-1 24000074 PMC3843776

[36] Papagelopoulos PJ Savvidou OD Koutsouradis P Chloros GD Diamantopoulos P. Three-dimensional technologies in orthopedics. Orthopedics. (2018) 41(1):12–20. 10.3928/01477447-20180109-04 29401368

[37] Park J Kang H Lim K Kim J Kim H. Three-dimensionally printed personalized implant design and re-constructive surgery for a bone tumor of the calcaneus: a case report. JBJS Case Connect. (2018) 8(2):e25. 10.2106/JBJS.CC.17.00212 29697440

[38] Imanishi J Choong PFM. Three-dimensional printed calcaneal prosthesis following total calcanectomy. Int J Surg Case Rep. (2015) 10:83–7. 10.1016/j.ijscr.2015.02.037 25827294 PMC4429954

